# Early Protein Markers of Necrotizing Enterocolitis in Plasma of Preterm Pigs Exposed to Antibiotics

**DOI:** 10.3389/fimmu.2020.565862

**Published:** 2020-10-08

**Authors:** Yan-Nan Jiang, Tik Muk, Allan Stensballe, Duc Ninh Nguyen, Per Torp Sangild, Ping-Ping Jiang

**Affiliations:** ^1^School of Public Health, Sun Yat-sen University, Guangzhou, China; ^2^Department of Veterinary and Animal Sciences, Section for Comparative Paediatrics and Nutrition, University of Copenhagen, Frederiksberg, Denmark; ^3^Department of Health Science and Technology, Aalborg University, Aalborg, Denmark; ^4^Department of Neonatology, Rigshospitalet, Copenhagen, Denmark

**Keywords:** necrotizing enterocolitis (NEC), antibiotics, proteomics, ECM, lipid metabilism, immunity

## Abstract

**Background:** Most hospitalized preterm infants receive antibiotics in the first days of life to prevent or treat infections. Short-term, early antibiotic treatment may also prevent the microbiota-dependent gut inflammatory disorder, necrotizing enterocolitis (NEC). It remains a challenge to predict NEC, and a few early blood diagnostic markers exist. Using preterm pigs as model for infants, blood parameters and plasma proteins affected by early progression of NEC were profiled in preterm pigs subjected to oral, systemic, or no antibiotics after preterm birth.

**Methods:** Preterm newborn pigs were treated with saline (CON) or antibiotics (ampicillin, gentamicin, and metronidazole) given enterally (ENT) or parenterally (PAR), and fed formula for 4 days to induce variable microbiome-dependent sensitivities to NEC. The gut was collected for macroscopic scoring of NEC lesions and blood for hematology, blood biochemistry, and LC/MS-based plasma proteomics. Statistical modeling was applied to detect plasma proteins affected by NEC and/or antibiotics.

**Results:** Analyzed across different antibiotic regimens, NEC progression was associated with altered blood parameters and abundance of 89 plasma proteins that were functionally involved in extracellular membrane destruction, lipid metabolism, coagulopathy, and acute phase response. Large NEC-related changes were observed in abundance of RBP4, FGA, AHSG, C5, PTPRG, and A-1-antichymotrypsin 2, indicating potential serving as early markers of NEC. Conversely, antibiotic treatment, independent of NEC, affected only 4 proteins with main differences found between ENT and CON pigs.

**Conclusion:** Early postnatal development of NEC lesions is associated with marked plasma protein changes that may be used for early NEC diagnosis.

## Introduction

Necrotizing enterocolitis (NEC) is a common gastrointestinal tract (GIT) disease with high mortality in preterm infants (1). Besides the gut symptoms, such as elevated permeability, immune cell infiltration, and tissue inflammatory response (1), NEC is closely related to systemic inflammation, potentially leading to injury of organs distant to the GIT, such as the brain and lungs (2). NEC-associated systemic inflammation includes changes in blood cell composition, such as leukopenia, monocytopenia, thrombocytopenia, and/or suppression of erythropoiesis, (3) and in plasma levels of pro-inflammatory cytokines (IL-6 and IL-8) (4) and multiple immune-related proteins, such as C-reactive protein (CRP), procalcitonin, and serum amyloid-A (SAA). All these are potential markers for NEC (5), but it remains difficult to differentiate NEC from systemic inflammatory conditions, like sepsis, which may be associated with NEC or occur independently. There is a need to better understand how gut inflammatory conditions may affect plasma proteins that could serve to predict NEC early, thus allowing timely NEC prevention and treatment (6).

The gut bacterial colonization in early life is involved in NEC. Dyscolonization with a few (pathogenic) strains may predispose to both NEC and systemic infections (7). Early antibiotic treatment, commonly used to treat or prevent sepsis and infection (8), affects the gut microbiome, and a less diverse gut microbiome is associated with NEC in preterm infants (9). Prolonged antibiotic treatment increases the incidence of NEC and sepsis (10, 11), but short-term systemic antibiotic treatment, given to about 90% of very preterm infants, is recently shown to be associated with less NEC in a survey from 13 NICUs across the world (12). This supports findings from previous studies demonstrating protection against NEC after prophylactic enteral antibiotics in infants (13) and preterm pigs (6, 14). In these studies, the enteral antibiotic treatment reduced gut bacterial load and diversity, and prevented structural and functional damage, hypoxic stress, and immune-related DNA methylation changes in the small intestinal tissue (6, 15). As reported earlier, NEC lesions observed in such preterm formula-fed pigs on day 5 of life are generally evident by macroscopic tissue inspection without any previous clinical signs of NEC, e.g., abdominal distention, bloody stools, apnea or lethargy, hence, representing the early phase of clinical NEC (6). Of note, the enteral antibiotic treatment also affected the systemic innate immunity (16), indicating that the antibiotic treatment may affect systemic parameters including plasma proteins, independent of the NEC effects. Among different biofluids available for disease biomarkers, blood remains the sample of choice due to its easy availability and its potential to reflect pathophysiological changes in a variety of organs.

On this background, we hypothesize that early postnatal progression of NEC, as detected in preterm pigs fed formula, induces plasma proteome changes reflecting systemic effects of early NEC. Considering the variable, but frequent, use of antibiotic treatment for preterm infants immediately after birth, and the critical role of the gut microbiome in NEC, preterm newborn pigs were exposed to either no antibiotics, systemic or enteral antibiotics in clinically relevant doses, creating a range of antibiotic-dependent NEC sensitivities. NEC-related systemic responses in these pigs were assessed by hematology, blood biochemistry, and plasma protein profile by mass spectrometry (MS)-based proteomics. Gene expression of selected plasma proteins affected by NEC or the antibiotic treatment was assessed in the liver and small intestinal tissue.

## Materials and Methods

### Animal Procedure and Antibiotic Treatment

Delivery, rearing, feeding, and antibiotic treatment were carried out as previously described (6). In brief, 47 preterm pigs were delivered from three sows (Large White × Danish Landrace × Duroc) by cesarean section on day 106 (90–92%) of gestation (day 1). After being fitted with umbilical arterial catheters (infant feeding tube 4F; Portex, Kent, UK) and orogastric feeding tubes (6F Portex), these pigs were reared in temperature- and oxygen-regulated incubators. A group of pigs was given antibiotics through the umbilical catheter (PAR, *n* = 17), the other 15 pigs received antibiotics via the orogastric tube (ENT, *n* = 15), and the remaining pigs received saline, serving as untreated controls (CON, *n* = 15). The antibiotics used were ampicillin (30 mg/kg BW, 3 times daily), gentamicin (2.5 mg/kg BW, twice daily), and metronidazole (10 mg/kg BW, 3 times daily), specifically formulated for enteral and parenteral use. The antibiotic treatment started immediately after the enteral feeding started on day 1 until the euthanasia on day 5. All pigs were given both parenteral nutrition (4 mL/kg/h in the first 24 h, gradually increasing to 6–8 mL/kg/h) and minimal enteral nutrition (3 mL/kg every 3 h) on days 1 and 2, before being shifted to full enteral feeding (15 mL/kg every 3 h) on day 3 until the end of the experiment on day 5. Formulations of both parenteral and enteral nutrition are provided as Supplementary Table 1.

On day 5, under anesthesia, all pigs were euthanized by an overdose of pentobarbital after blood sampling through an intracardiac puncture. Whole blood was collected for cell counting, and EDTA-treated plasma was saved for blood biochemistry and proteomic analysis. As previously described (14), each pig was given an oral bolus (15 mL/kg BW) of a solution containing 5% lactulose and 5% mannitol 3 h before the planned euthanasia, and a urine sample was collected via cystocentesis at euthanasia. Prior to the oral bolus, individual pigs randomly underwent a 2 to 4 h fasting period, and received the last enteral feeding 60 min before the urine collection following euthanasia. Intestinal permeability was assessed by the urinary ratio of lactulose and mannitol. The GIT of each piglet was collected, and five regions, namely, the stomach, proximal, middle, and distal small intestines, and colon, were separately evaluated for macroscopic NEC severity using a validated NEC scoring system as follows: (1) absence of macroscopic hemorrhage, edema, or mucosal abnormality; (2) local hyperemia; (3) hyperemia, extensive edema and local hemorrhage; (4) extensive hemorrhage; (5) local necrosis and pneumatosis intestinalis; and (6) extensive transmural necrosis and pneumatosis intestinalis (14). The maximal NEC score across these five regions was recorded as the NEC score of the pig to indicate the overall NEC severity.

Blood cell counting was conducted on an Advia 2120i Hematology System (Siemens, Munich, Germany). Plasma biochemistry was analyzed using Advia 1800 Chemistry systems (Siemens, Erlangen, Germany). The study was approved by the Danish National Committee of Animal Experimentation (no. 2014-15-0201-00418).

### LC/MS-Based Plasma Proteomics

The preparation of a protein sample was performed using a filter-aided protocol, as previously described (17). Briefly, protein concentration in the plasma samples was determined on a NanoDrop Spectrophotometer (Thermo Scientific, Waltham, MA, USA). Plasma sample containing 100 μg protein was transferred onto an Amicon Ultra centrifugal filter (10 kDa, 0.5 mL, Millipore, Søborg, Denmark), and mixed with a buffer containing sodium deoxycholate (5%) and triethylammonium bicarbonate (50 mmol/L, pH 8.0). Protein was reduced by TCEP solution [0.01 mol/L, 1:50 (v/v)], alkylated by chloroacetamide [0.5 mol/L, 1:50 (v/v)], and digested by trypsin (Promega, 1 μg/100 μg protein, 37°C overnight) inside the spin filter with a centrifuge step (14,000 × g for 15 min) in between. Tryptic peptides were recovered by another step of centrifugation and purified by phase extraction using ethyl acetate acidified by trifluoroacetic acid (1%, v/v). Vacuum-dried peptides were suspended in a solution of 2% acetonitrile, 0.1% formic acid, and 0.1% trifluoroacetic acid, and applied onto a Dionex RSLC UPLC System (Thermo Scientific) coupled to a Q-Exactive HF Hybrid Quadrupole-Orbitrap Mass Spectrometer (Thermo Scientific). Five micrograms of peptide was injected onto a 2 cm reverse-phase C18 material-trapping column and separated on a 50-cm analytical column (Acclaim PepMap100, 75 μm ID, 100 Å, Thermo Scientific) with both columns kept at 40°C. Elution gradient at a constant flow rate of 300 nl/min started with a mixture of water (97.9%) and acetonitrile (2%) containing 0.1% formic acid, then increased to 30% acetonitrile in 225 min. Mass spectrometric data were obtained in positive ionization mode in a data-dependent acquisition (DDA) fashion with survey spectra and isolation/fragmentation spectra alternating using a Top12 method. Selected peptides were excluded from reanalysis for 30 s.

Protein annotation and quantification based on mass spectra of peptides were carried out using MaxQuant (1.5.2.8) (18) against the Uniprot reference database with isoforms (*Sus scrofa*, UP000008227, last modified 2016-08-02). Detection of at least two unique peptides per protein and protein being present in at least 70% of the samples in each group were required for protein annotation and quantification. Protein abundance data were normalized and two-based logarithm transformed using the Perseus software (version 1.2.0.17) (19), then aligned with protein identities and grouping information, such as treatment, litter (sow), NEC score, and sex, and exported into R (version 3.4.1) (20) integrated with R Studio (version 3.1.18) (21) for data analysis. The MS proteomics data are available at the ProteomeXchange Consortium (http://www.proteomexchange.org/) with the data set identifier PXD015938.

### RT-qPCR of Hepatic and Distal Small Intestinal Genes

To balance the effect of litter and sex for treatment comparisons, one pig of each sex from each litter was selected for each treatment group. A random number selection method was used to choose the sample when more than one pig was eligible for each litter, sex, and treatment. Two more pigs (one male and one female) were randomly selected from any two treatment groups with eligible candidates, resulting in total 24 pigs selected (*n* = 8 in each group) for the RT-qPCR analysis. The NEC scores of the selected pigs were not significantly different from those of the entire groups (χ^2^ test, *P* = 0.90). Transcription of selected genes in the liver and distal small intestine was determined by RT-qPCR, using predesigned primers (sequences listed in Supplementary Table 3). Briefly, total RNA in the tissue homogenate was isolated with RNeasy Lipid Tissue Mini Kit (Qiagen, Copenhagen, Denmark). RT-qPCR was performed using QuantiTect SYBR Green PCR Kit (Qiagen) on a LightCycler 480 (Roche, Hvidovre, Denmark). Levels of target gene were normalized to that of the housekeeping gene, HPRT1 (22), before further statistical analysis.

### Data Analysis

Univariate analysis was applied to hematologcial, blood biochemical, and proteomic data. A linear mixed-effect model with the antibiotic treatment (CON, PAR, and ENT), NEC score in continuous mode, and sex of the pig as fixed-effect factors, while litter (sow) being a random-effect factor, was fitted to each parameter (hematology, blood biochemistry, and proteomics) using the nlme package (23). Variance Inflation Factor (VIF) of the model was tested by the *vif* function to evaluate the possible colinearity of treatment and NEC score. A VIF larger than 2.5 indicated existence of colinearity, and the model would be rejected. The effect of treatment or NEC was tested by comparing this model with another model without treatment or NEC score as factor, respectively. The difference between the treatment levels was tested in a pairwise fashion by the Tukey *post hoc* test (package multcomp). The regression coefficient of NEC severity was used to show the effect of NEC severity on each parameter. To control the type I error of analysis of the proteomics data, the *P*-value obtained was further adjusted by false discovery rate (FDR, α = 0.2) into q-value using the multtest package (24). Proteins with a value of *q* ≤ 0.10 in any comparisons between the treatment groups were selected for functional assignment.

To explore associations between proteins revealed by the proteomic analysis, their abundance was applied to Spearman correlation analysis in pairwise fashion. Correlations with the absolute value of Spearman's r <0.7 were manually clustered and imported into AutoAnnotate (Version 1.3.3) (25) based on Cytoscape (Version 3.8.0) (26) to generate a protein correlation network.

Results from RT-qPCR were analyzed using Student's *t*-test, and a two-tailed *P* < 0.05 was considered as statistically significant.

## Results

### Clinical Data, Hematology, and Blood Biochemistry

**Figure 1:**
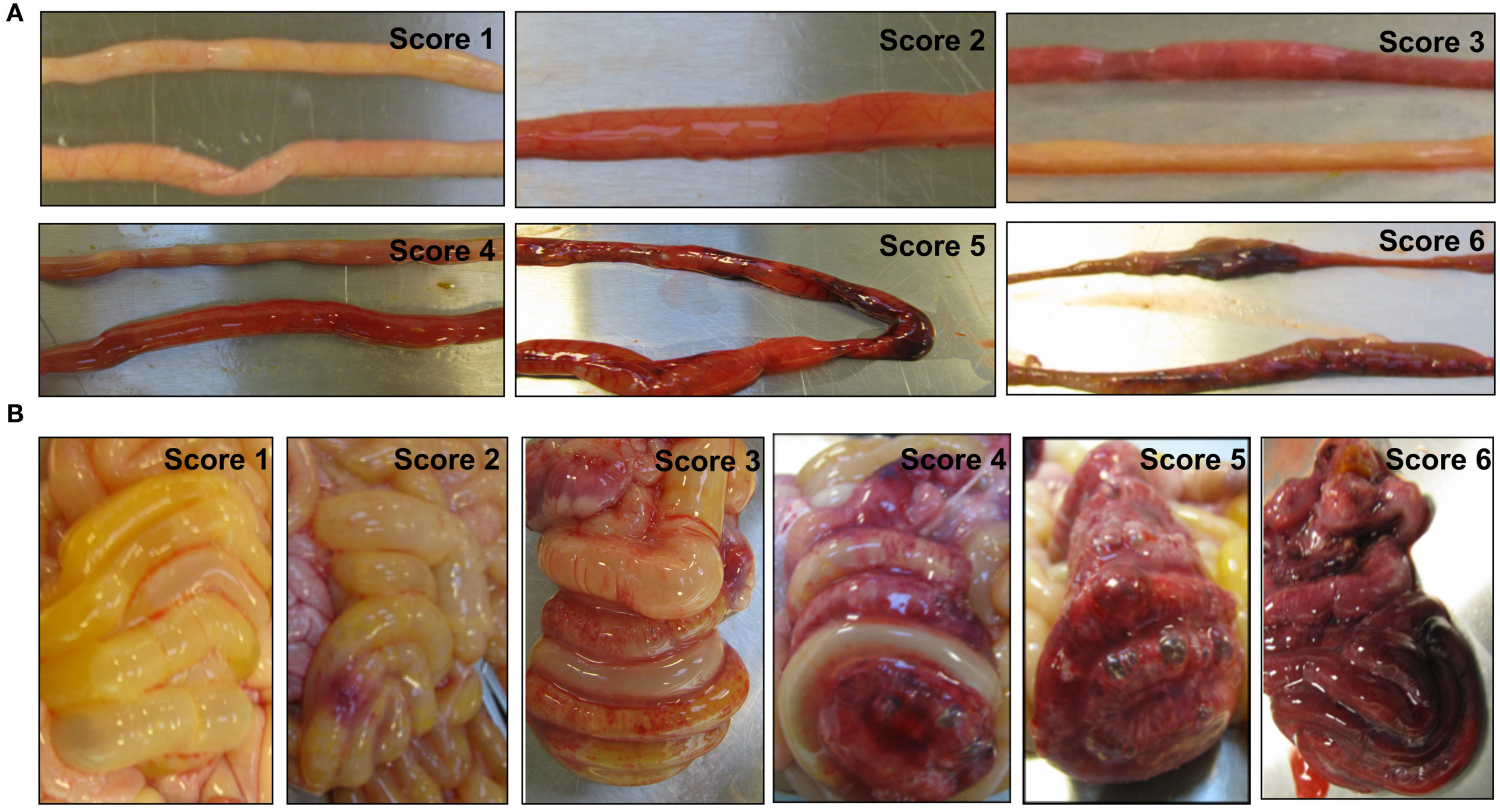
Representative images of the small intestine **(A)** and the colon **(B)** with or without necrotizing enterocolitis (NEC) lesions. The macroscopic NEC severity was evaluated using a scoring system as follows: 1, absence of macroscopic hemorrhage, edema, or mucosal abnormality; 2, local hyperemia; 3, hyperemia, extensive edema, and local hemorrhage; 4, extensive hemorrhage; 5, local necrosis and pneumatosis intestinalis; and 6, extensive transmural necrosis and pneumatosis intestinalis.

A total of 47 pigs out of the initial 64 pigs were included in this study, while 17 pigs dying within the first 2 days from immaturity-related complications (respiratory distress and immaturity of lungs) were excluded. Hematological and plasma biochemical parameters of the pigs included for the proteomic analysis are listed in Table 1, and NEC scores of each treatment group are listed in Supplementary Table 2 and Supplementary Figure 1. NEC severity of the small intestine and colon was scored according to their NEC lesions, and representative images are displayed in Figure 1. Lower NEC scores were found in ENT pigs, relative to both PAR and CON pigs (two-tailed *t*-test, *P* < 0.05). PAR pigs also had lower NEC score than CON pigs (*P* < 0.05). Regardless of NEC, significantly lower monocyte numbers (absolute counts or relative percentage, both *P* < 0.05) found in the antibiotic groups (PAR or ENT), had no significant difference between the two groups. ENT pigs had the lowest number of neutrophils (*P* < 0.05, ENT vs. CON). The antibiotic treatment tended to reduce levels of total plasma protein (PAR vs. CON, *P* < 0.05; ENT vs. CON, *P* = 0.08) and albumin (PAR vs. CON, *P* = 0.05).

**Table 1:** Hematology and blood biochemistry.

	**Abundance^a^ by treatment**			***P*****-value**			**NEC severity**	
	**CON**	**PAR**	**ENT**	**PAR- CON**	**ENT- CON**	**ENT- PAR**	**Coefficient^b^**	***P*-value**
**HEMATOLOGY**								
WBC (10^9^/L)	2.3 ± 0.4	2.2 ± 0.2	2.2 ± 0.2	0.54	0.17	0.56	−0.31	<0.01
Neutrophils (10^9^/L)	0.80 ± 0.21	0.72 ± 0.09	0.59 ± 0.06	0.63	0.04	0.13	−0.13	0.02
Lymphocytes (10^9^/L)	1.31 ± 0.14	1.38 ± 0.12	1.54 ± 0.12	0.94	0.97	1	−0.16	0.01
Monocytes (10^9^/L)	0.11 ± 0.03	0.06 ± 0.01	0.06 ± 0.01	0.01	<0.01	0.28	−0.02	0.01
Basophils (10^9^/L)	0.01 ± 0.00	0.01 ± 0.00	0.01 ± 0.00	0.72	0.20	0.52	<0.01	0.46
Eosinophils (10^9^/L)	0.02 ± 0.00	0.01 ± 0.00	0.01 ± 0.00	0.38	0.26	0.88	<0.01	0.78
Neutrophils (%)	31.4 ± 3.6	31.0 ± 2.6	26.3 ± 2.0	0.97	0.27	0.11	−0.69	0.54
Lymphocytes (%)	61.0 ± 4.0	63.5 ± 2.5	68.6 ± 2.3	0.95	0.10	0.10	0.77	0.52
Monocytes (%)	4.5 ± 0.6	3.0 ± 0.3	2.7 ± 0.5	0.03	0.02	0.82	−0.10	0.63
Basophils (%)	0.5 ± 0.1	0.4 ± 0.1	0.3 ± 0.0	0.95	0.97	1	0.05	0.16
Eosinophils (%)	0.6 ± 0.2	0.4 ± 0.1	0.4 ± 0.1	0.53	0.74	0.98	0.04	0.43
Erythrocytes (10^12^/L)	4.0 ± 0.2	4.0 ± 0.2	4.0 ± 0.2	0.79	0.77	0.99	−0.06	0.38
Hemoglobin (mmol/L)	4.9 ± 0.2	4.9 ± 0.2	4.9 ± 0.2	0.77	0.80	1	−0.06	0.49
Hematocrit (%)	26.4 ± 1.2	26.2 ± 1.0	26.5 ± 1.1	0.72	0.82	1	−0.32	0.51
MCV (fl)	65.4 ± 0.9	66.0 ± 0.5	66.7 ± 0.6	0.80	0.69	0.95	−0.12	0.66
MCHC (g/dl)	18.5 ± 0.1	18.6 ± 0.1	18.4 ± 0.1	0.88	0.71	0.35	−0.01	0.77
Thrombocytes (10^9^/L)	137.2 ± 15.2	124.9 ± 18.7	106.9 ± 18.3	0.96	0.34	0.36	−11.4	0.11
MPV (fl)	8.5 ± 0.4	8.1 ± 0.3	8.3 ± 0.2	0.35	0.38	0.98	−0.17	0.20
MPC (g/dl)	209.6 ± 4.2	213.4 ± 4.1	217.3 ± 4.2	0.93	0.19	0.22	2.5	0.16
**BLOOD BIOCHEMICAL PARAMETERS**								
Total protein, g/L	29.5 ± 0.7	27.6 ± 0.5	27.5 ± 0.4	0.03	0.08	1	0.01	0.97
Albumin, g/L	12.5 ± 0.4	11.6 ± 0.3	11.6 ± 0.2	0.05	0.15	0.99	<0.01	0.93
ALT, U/L	19.9 ± 1.5	20.2 ± 1.9	17.5 ± 0.6	0.77	0.94	0.96	1.4	0.01
AST, U/L	46.1 ± 10.8	93.8 ± 36.2	55.6 ± 30.1	0.19	0.38	0.99	22.8	0.03
ALP, 10^3^ U/L	3.1 ± 0.3	2.8 ± 0.2	2.6 ± 0.3	0.89	0.95	1	0.16	0.10
GGT, U/L	26.9 ± 4.1	25.9 ± 4.0	21.7 ± 2.2	0.86	0.45	0.70	3.3	0.01
Bilirubin, μmol/L	2.0 ± 0.7	1.1 ± 0.4	0.5 ± 0.1	0.47	0.87	0.87	0.62	<0.01
Total cholesterol, mmol/L	2.4 ± 0.1	2.4 ± 0.2	2.6 ± 0.1	0.79	0.55	0.86	−0.14	<0.01
Urea, mmol/L	10.3 ± 0.7	10.1 ± 0.7	10.0 ± 1.0	0.43	0.08	0.48	−0.69	0.03
Creatinine, μmol/L	56.0 ± 3.5	56.2 ± 3.0	47.0 ± 1.6	0.95	0.19	0.08	0.05	0.88
Creatine kinase, U/L	166.1 ± 33.4	317.2 ± 95.3	224.4 ± 109.8	0.25	0.47	0.98	48.2	0.15
Iron, μmol/L	6.3 ± 1.0	5.3 ± 0.5	8.0 ± 0.9	0.87	0.57	0.28	−0.25	0.53
Ionized phosphate, mmol/L	1.2 ± 0.1	1.4 ± 0.2	1.2 ± 0.1	0.19	0.30	1	0.14	0.01
Ca, mmol/L	3.0 ± 0.0	3.0 ± 0.0	3.0 ± 0.0	0.44	0.70	0.97	−0.03	0.03
Mg, mmol/L	0.9 ± 0.0	0.9 ± 0.0	0.9 ± 0.0	0.38	0.54	1	0.02	0.28
Na, mmol/L	158.1 ± 1.4	157.9 ± 1.1	161.0 ± 2.0	0.96	0.67	0.47	−0.30	0.70
K, mmol/L	4.5 ± 0.1	4.9 ± 0.5	5.6 ± 1.3	0.84	0.23	0.42	0.46	0.22
**INTESTINAL PERMEABILITY**								
Lactulose/mannitol ratio (10^−2^)	8.8 ± 3.3	9.5 ± 2.7	3.8 ± 1.3	0.55	<0.01	0.01	−2.4	0.02

a *Data are shown as mean ± SEM*.

b *Regression coefficient from the linear mixed-effect model indicating the effect of NEC severity. CON, no antibiotic treatment; PAR, parenteral antibiotics administered; ENT, enteral antibiotics administered*.

*WBC, total leukocytes; MCV, mean corpuscular volume; MCHC, mean corpuscular hemoglobin concentration; MPV, mean platelet volume; MPC, mean platelet component; ALP, alkaline phosphatase; ALT, alanine transaminase; AST, aspartate aminotransferase; GGT, γ-glutamyltransferase*.

NEC severity, as indicated by NEC scores, negatively affected the numbers of immune cells (total white blood cells, neutrophils, lymphocytes, and monocytes) (Table 1). Conversely, blood biochemical parameters reflecting liver (dys)functions increased with increasing NEC score (ALP, ALT, bilirubin, AST, and GGT, all Ps < 0.05). Furthermore, NEC severity scores negatively affected the cholesterol, carbamide and calcium levels (all Ps < 0.05) (Table 1). Intestinal permeability, as indicated by the ratio of urinary levels of lactulose over mannitol, increased with increasing NEC severity (*P* < 0.01) (Table 1).

### Plasma Proteomics

**Figure 2:**
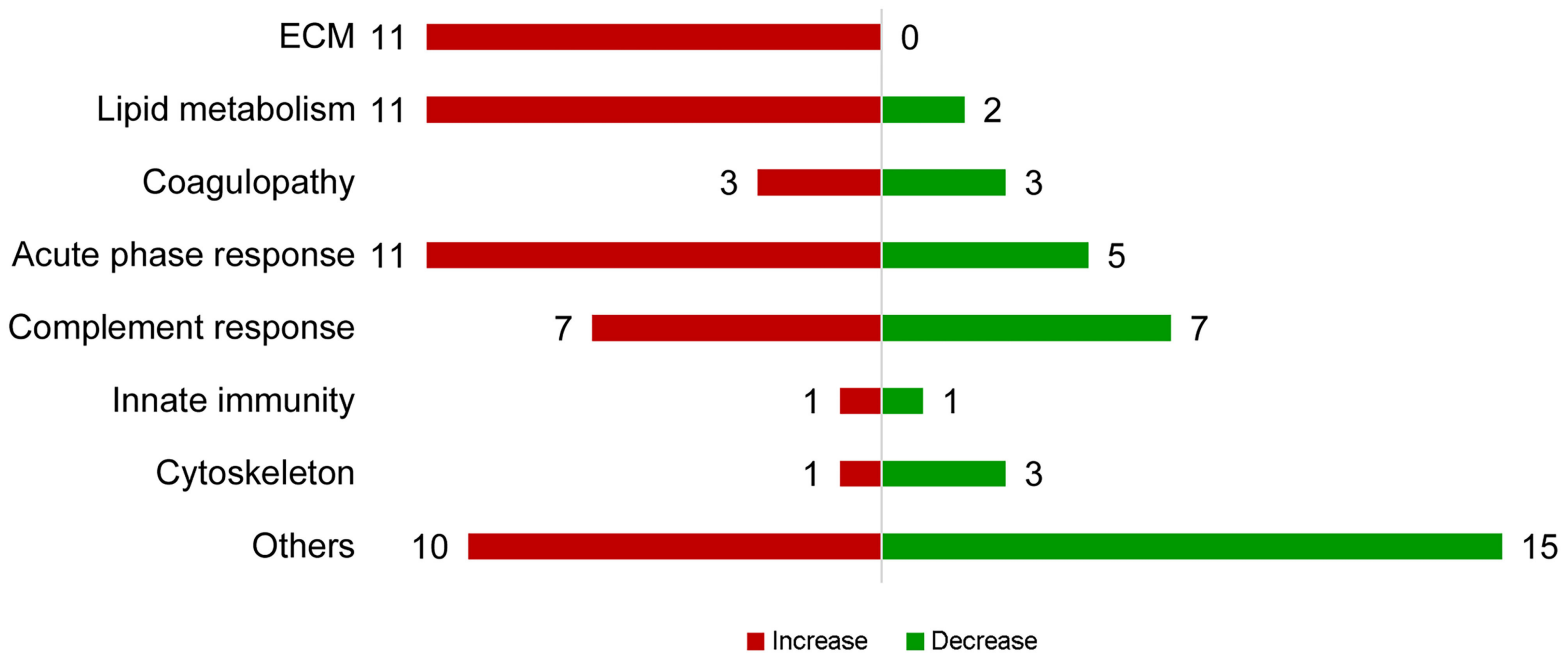
Summary of proteins with abundance changing with NEC severity. Proteins with increasing abundance are in red, decreasing ones in green.

In total, 303 plasma proteins were successfully annotated. Information of proteins with differential abundance, including UniProt ID, gene name, protein name, and abundance in each antibiotic treatment group or regression coefficient of NEC severity, is listed in functional groups in Table 2. None of the statistical models, testing the effect of the antibiotic treatment and NEC, had a VIF above 2.5 indicating that keeping both treatment and NEC severity in these models does not inflate the variance; thus, testing the effect of both factors is reliable. Results showed that 90 proteins were significantly affected (*q* ≤ 0.10) by either the antibiotic treatment or NEC. Among the differential proteins, only four proteins, namely, serpin, a6 and a8, angiotensinogen, and complement factor I (CFI), were significantly affected by the antibiotic treatment, with changes mainly observed between ENT and untreated control pigs (*q* ≤ 0.10), except for CFI (ENT vs. PAR, *q* = 0.08). In contrast, increasing NEC score was associated with changed abundance of 89 plasma proteins. These proteins are involved in several biological processes, including extracellular matrix (ECM) homeostasis, lipid metabolism, coagulopathy, innate immunity, and cytoskeleton. Direction of change in protein levels is summarized in Figure 2.

**Table 2:** Proteins with differential abundance by NEC or the antibiotic treatment.

**Uniprot ID**	**Gene**	**Protein**	**Abundance^a^ by treatment**			**q-value**			**NEC severity**	
			**CON**	**PAR**	**ENT**	**PAR- CON**	**ENT- CON**	**ENT- PAR**	**Coefficient^b^**	**q-value**
**PROTEINS AFFECTED BY ANTIBIOTICS TREATMENT**										
F1RG45	AGT	Angiotensinogen preproprotein	29.2 ± 0.2	29.4 ± 0.2	29.5 ± 0.2	0.77	0.08	0.99	0.32	<0.01
CBG	Serpina6	Corticosteroid-binding globulin	26.7 ± 0.1	26.9 ± 0.2	27.3 ± 0.1	0.97	0.08	0.12	0.07	0.18
F1S133	CFI	Complement factor I	28.1 ± 0.1	27.9 ± 0.1	28.3 ± 0.1	0.86	0.39	0.06	0.05	0.10
F1SCD0	LOC100153899	Serpin A3-8	32.9 ± 0.2	33.2 ± 0.2	33.2 ± 0.1	0.45	0.06	0.99	0.23	<0.01
**PROTEINS AFFECTED BY NEC**										
**EXTRACELLULAR MATRIX HOMEOSTASIS**										
F1RFU7	CDH11	Cadherin-11 isoform X1	22.6 ± 0.2	22.8 ± 0.2	23.0 ± 0.1	0.97	0.98	0.99	−0.19	0.05
F1S021	COL5A1	Collagen α-1(V) chain	24.4 ± 0.1	24.7 ± 0.2	24.9 ± 0.2	0.97	0.98	0.99	−0.21	0.02
I3LUR7	COL6A3	Collagen type VI α 3 chain	26.6 ± 0.1	26.9 ± 0.1	27.1 ± 0.1	0.97	0.96	0.99	−0.12	0.05
F1RTT3	COL9A1	Collagen α-1(V) chain	22.8 ± 0.2	23.0 ± 0.3	23.3 ± 0.2	0.97	0.98	0.99	−0.26	0.02
F1S902	COMP	Cartilage oligomeric matrix protein	24.3 ± 0.1	24.2 ± 0.1	24.6 ± 0.1	0.97	0.98	0.99	−0.14	0.02
I3LC64	ECM1	Extracellular matrix protein 1	25.3 ± 0.2	25.3 ± 0.2	25.3 ± 0.2	0.97	0.78	0.99	−0.20	0.02
F1SQL2	EFEMP1	EGF containing fibulin extracellular matrix protein 1	23.2 ± 0.1	23.5 ± 0.1	23.5 ± 0.1	0.97	0.98	0.99	−0.12	0.06
F1SMF4	ITGA2	Integrin subunit α-2	22.4 ± 0.1	22.8 ± 0.2	23.1 ± 0.1	0.97	0.96	0.99	0.16	0.03
F1RF11	MMP2	72 kDa type IV collagenase	22.9 ± 0.2	23.4 ± 0.2	23.8 ± 0.1	0.87	0.51	0.99	−0.17	0.06
F1S682	QSOX1	Sulfhydryl oxidase	28.1 ± 0.1	28.1 ± 0.1	28.2 ± 0.1	0.97	0.98	0.99	−0.06	0.10
VTNC	VTN	Vitronectin	25.5 ± 0.3	25.8 ± 0.4	26.5 ± 0.2	0.97	0.98	0.99	−0.27	0.06
**LIPID METABOLISM**										
APOA4	SAA2	Serum amyloid A protein	32.0 ± 0.1	32.3 ± 0.1	32.5 ± 0.1	0.64	0.59	0.99	−0.10	0.01
D3Y264	APOA4	Apolipoprotein A-IV	28.4 ± 0.2	28.9 ± 0.2	28.7 ± 0.2	0.89	0.98	0.99	−0.24	0.01
APOC3	APOC2	Apolipoprotein C-II	31.4 ± 0.2	31.4 ± 0.2	31.9 ± 0.1	0.97	0.98	0.99	−0.26	<0.01
F1SQX9_	APOC3	Apolipoprotein C-III	29.3 ± 0.2	29.3 ± 0.2	29.5 ± 0.2	0.97	0.98	0.99	−0.26	0.01
APOE	APOD	Apolipoprotein D	30.4 ± 0.1	30.3 ± 0.1	30.6 ± 0.1	0.97	0.98	0.99	−0.09	0.09
Q68RU1	APOE	Apolipoprotein E	24.6 ± 0.4	24.6 ± 0.3	24.8 ± 0.3	0.97	0.75	0.99	0.30	0.07
Q4Z8N7	ApoN	Ovarian and testicular apolipoprotein N	24.9 ± 0.2	25.3 ± 0.2	25.6 ± 0.1	0.97	0.98	0.99	−0.20	0.02
I3LGB2	PAF-AH	Platelet-activating factor acetylhydrolase	24.7 ± 0.1	24.6 ± 0.2	24.8 ± 0.1	0.97	0.98	0.99	−0.19	0.01
F1SC57	PCSK9	Proprotein convertase subtilisin/kexin type 9	24.2 ± 0.1	24.2 ± 0.1	24.3 ± 0.2	0.97	0.98	0.99	−0.11	0.06
F1SFA1	PLTP	Phospholipid transfer protein	24.8 ± 0.1	24.9 ± 0.2	25.2 ± 0.1	0.97	0.98	0.99	−0.16	0.03
RET4	PON1	Paraoxonase 1	27.2 ± 0.1	26.9 ± 0.2	27.4 ± 0.1	0.42	0.97	0.99	−0.19	0.01
F1S9B9	RBP4	Retinol-binding protein 4	23.4 ± 0.6	23.7 ± 0.9	21.6 ± 0.9	0.97	0.98	0.99	0.84	0.03
TTHY	TTR	Transthyretin	31.9 ± 0.1	32.0 ± 0.1	32.1 ± 0.1	0.97	0.98	0.99	−0.08	0.06
**COAGULOPATHY**										
FA5	F5	Coagulation factor V	27.9 ± 0.2	27.9 ± 0.2	28.2 ± 0.1	0.97	0.98	0.99	−0.22	<0.01
FIBA	FGA	Fibrinogen-α-chain	28.5 ± 0.6	28.2 ± 0.6	27.0 ± 0.4	0.97	0.98	0.99	0.62	0.02
F1S5J5	HABP2	Hyaluronan binding protein 2	23.4 ± 0.1	23.3 ± 0.1	23.5 ± 0.1	0.97	0.43	0.99	0.08	0.10
F1SFI5	HRG	Histidine-rich glycoprotein	31.0 ± 0.2	31.4 ± 0.1	31.2 ± 0.1	0.51	0.43	0.99	0.13	0.08
F1SK70	PROS1	Vitamin K-dependent protein S isoform 2 preproprotein	27.9 ± 0.1	28.0 ± 0.0	28.2 ± 0.1	0.97	0.66	0.99	−0.05	0.06
F2Z5E2	SERPINC1	Antithrombin III	31.7 ± 0.1	31.7 ± 0.1	31.9 ± 0.1	0.97	0.96	0.99	−0.13	<0.01
**ACUTE PHASE RESPONSE**										
F1RG45	AGT	Angiotensinogen preproprotein	29.2 ± 0.2	29.4 ± 0.2	29.5 ± 0.2	0.77	0.08	0.99	0.32	<0.01
FETUA	AHSG	A-2-HS-glycoprotein	34.4 ± 0.3	33.8 ± 0.4	34.2 ± 0.4	0.57	0.30	0.99	−0.53	0.01
ALBU	ALB	Serum albumin	34.9 ± 0.2	34.5 ± 0.2	34.9 ± 0.2	0.28	0.35	0.99	−0.30	<0.01
AMBP	AMBP	Protein AMBP	28.4 ± 0.2	27.8 ± 0.2	28.4 ± 0.2	0.13	0.62	0.99	−0.20	0.03
F1SKB1	CP	Ceruloplasmin	30.2 ± 0.2	29.5 ± 0.1	29.5 ± 0.1	0.13	0.35	0.99	0.12	0.07
F1S8V7	CPN1	Carboxypeptidase N catalytic chain	25.7 ± 0.2	25.6 ± 0.1	25.7 ± 0.2	0.80	0.98	0.99	−0.11	0.09
F1SH96	ITIH1	Inter-α-trypsin inhibitor heavy chain H1	28.2 ± 0.1	28.2 ± 0.1	28.6 ± 0.1	0.97	0.98	0.99	−0.18	<0.01
ITIH1	ITIH1	Inter-α-trypsin inhibitor heavy chain H1	29.0 ± 0.2	28.6 ± 0.2	29.1 ± 0.1	0.13	0.30	0.99	−0.24	<0.01
ITIH2	ITIH2	Inter-α-trypsin inhibitor heavy chain H2	31.1 ± 0.1	31.1 ± 0.1	31.4 ± 0.1	0.97	0.98	0.99	−0.17	<0.01
F1SH92	ITIH4	Inter-α-trypsin inhibitor heavy chain H4	31.9 ± 0.2	31.6 ± 0.1	31.5 ± 0.1	0.76	0.98	0.99	0.12	0.03
I3L5U6	LBP	Lipopolysaccharide binding protein	25.5 ± 0.2	24.8 ± 0.2	24.7 ± 0.2	0.68	0.98	0.99	0.21	0.02
F1SN68	ORM1	A-1-acid glycoprotein	34.9 ± 0.1	34.8 ± 0.1	34.9 ± 0.1	0.97	0.83	0.99	−0.09	0.05
Q9GMA6	SERPINA3-2	A-1-antichymotrypsin 2	30.5 ± 0.2	30.9 ± 0.2	30.7 ± 0.2	0.41	0.17	0.99	0.28	<0.01
TRFE	TF	Serotransferrin	34.4 ± 0.2	34.3 ± 0.1	34.5 ± 0.2	0.97	0.98	0.99	−0.16	0.06
**COMPLEMENT SYSTEM**										
F1SLV6	MASP1	Complement component MASP3	26.4 ± 0.2	26.0 ± 0.1	26.2 ± 0.2	0.47	0.31	0.99	−0.17	0.03
F1RQW7	C1R	Complement c1r	23.4 ± 0.3	22.9 ± 0.3	22.3 ± 0.2	0.97	0.98	0.99	0.37	<0.01
F1SBS4	C2	Complement C2	24.8 ± 0.7	25.5 ± 0.5	25.2 ± 0.6	0.82	0.35	0.99	0.71	0.01
I3LTB8	C3	Complement C3	26.1 ± 0.7	25.2 ± 0.9	24.2 ± 0.9	0.97	0.98	0.99	0.86	0.03
F1RQW2	C3	Complement C3	30.7 ± 0.1	30.5 ± 0.1	30.5 ± 0.1	0.71	0.43	0.99	−0.09	0.10
F1SME1	C4A	Complement C4-A isoform 1 preproprotein	28.2 ± 0.1	28.0 ± 0.2	27.9 ± 0.2	0.97	0.96	0.99	0.23	<0.01
F1SMI8	C5	Complement C5a anaphylatoxin	21.9 ± 0.5	20.6 ± 0.5	20.5 ± 0.4	0.97	0.98	0.99	0.48	0.01
F1S788	C6	Complement C6	26.3 ± 0.1	26.6 ± 0.1	26.7 ± 0.1	0.97	0.98	0.99	−0.18	<0.01
F1S790	C8A	Complement C8 α chain	26.9 ± 0.1	26.8 ± 0.1	27.2 ± 0.1	0.73	0.98	0.99	−0.16	<0.01
A0SEH3	C8B	Complement C8 β chain	25.8 ± 0.1	25.6 ± 0.1	25.7 ± 0.1	0.51	0.31	0.99	−0.15	0.01
F1S0J0	C8G	Complement component C8G	22.4 ± 0.1	22.5 ± 0.1	22.5 ± 0.1	0.97	0.98	0.99	−0.13	0.03
F1S133	CD55	Complement decay-accelerating factor	28.1 ± 0.1	27.9 ± 0.1	28.3 ± 0.1	0.86	0.39	0.06	0.05	0.10
D5L7X4	CFI	Complement factor I	22.4 ± 0.2	22.6 ± 0.2	23.0 ± 0.2	0.97	0.98	0.99	−0.18	0.09
F1SJW8	SERPING1	Plasma protease C1 inhibitor	29.3 ± 0.3	29.8 ± 0.2	29.7 ± 0.2	0.45	0.21	0.99	0.28	0.01
**INNATE IMMUNITY**										
F1SGT4	CD44	CD44 molecule	25.2 ± 0.1	25.2 ± 0.2	25.3 ± 0.2	0.97	0.84	0.99	−0.18	0.01
OSTP	SPP1	Osteopontin	24.3 ± 0.2	24.4 ± 0.2	24.2 ± 0.2	0.97	0.97	0.99	0.21	0.04
**CYTOSKELETON**										
I3L6D7	DSG2	Desmoglein 2	21.7 ± 0.3	21.9 ± 0.2	21.8 ± 0.3	0.95	0.46	<0.01	0.28	0.02
GELS	GSN	Gelsolin	29.7 ± 0.2	29.8 ± 0.2	30.0 ± 0.1	0.97	0.98	0.99	−0.20	0.01
F1RK02	LCP1	Lymphocyte cytosolic protein 1	22.3 ± 0.2	22.5 ± 0.2	22.5 ± 0.2	0.97	0.48	0.99	0.17	0.08
F1RFY1	PFN1	Profilin	21.9 ± 0.2	21.9 ± 0.3	21.6 ± 0.3	0.97	0.98	0.99	0.28	0.01
**OTHERS**										
F1RUM1	AFM	Afamin	28.8 ± 0.1	28.6 ± 0.1	28.8 ± 0.1	0.63	0.51	0.99	−0.17	0.01
FETA	AFP	A-fetoprotein	34.2 ± 0.1	34.0 ± 0.1	34.2 ± 0.1	0.35	0.51	0.99	−0.08	0.06
AMPN	ANPEP	Aminopeptidase N	21.6 ± 0.3	21.9 ± 0.2	21.9 ± 0.2	0.89	0.24	0.99	0.27	0.01
F1SBE4	B4GALT5	β-1,4-galactosyltransferase 5	23.0 ± 0.1	23.2 ± 0.1	23.2 ± 0.1	0.45	0.21	0.99	0.12	0.02
B9UJD6	C1QTNF3	C1q and tumor necrosis factor related protein 3 isoform b	22.0 ± 0.1	22.0 ± 0.2	22.2 ± 0.2	0.97	0.97	0.99	−0.24	0.01
I3LRD3	CENPE	Kinesin-like protein	24.8 ± 0.3	24.7 ± 0.3	25.1 ± 0.3	0.97	0.98	0.99	−0.27	0.08
F1SC70	CTSA	Carboxypeptidase	23.2 ± 0.1	23.3 ± 0.2	23.7 ± 0.2	0.97	0.17	0.99	0.11	0.09
F1SPE9	DNAJC13	Dnaj heat shock protein family (Hsp40) member C13	27.5 ± 0.3	27.9 ± 0.3	27.9 ± 0.3	0.76	0.21	0.99	0.40	0.01
I3LK59	ENO1	A-enolase isoform 1	21.7 ± 0.4	21.7 ± 0.5	21.7 ± 0.4	0.97	0.96	0.99	0.38	0.07
F1S715	FUCA2	A-L-fucosidase	25.1 ± 0.2	25.2 ± 0.2	25.6 ± 0.1	0.97	0.17	0.99	0.11	0.09
I3LN42	GC	Vitamin D-binding protein	32.0 ± 0.1	32.0 ± 0.1	32.1 ± 0.1	0.97	0.76	0.99	−0.13	0.02
F1S4I1	GOLM1	Golgi membrane protein 1	24.9 ± 0.4	25.0 ± 0.5	24.4 ± 0.3	0.97	0.98	0.99	0.41	0.03
GPX5	GPX5	Epididymal secretory glutathione peroxidase	25.3 ± 0.2	25.3 ± 0.1	25.6 ± 0.1	0.97	0.98	0.99	−0.17	0.03
F1SBR6	HIPK1	Homeodomain interacting protein kinase 1	22.3 ± 0.1	22.4 ± 0.1	22.5 ± 0.2	0.97	0.98	0.99	−0.13	0.05
F1SJL1	IGDCC4	Immunoglobulin superfamily DCC subclass member 4	22.4 ± 0.2	22.7 ± 0.1	22.6 ± 0.1	0.97	0.98	0.99	−0.18	0.01
F1SCC6	LOC100153899	Serpin A3-8	32.3 ± 0.3	31.1 ± 0.3	30.6 ± 0.3	0.48	0.89	0.99	0.46	<0.01
F1SCD0	LOC100153899	Serpin A3-8	32.9 ± 0.2	33.2 ± 0.2	33.2 ± 0.1	0.45	0.06	0.99	0.23	<0.01
F1SCC9	LOC106504545	Serpin A3-8	29.7 ± 0.4	30.4 ± 0.2	30.3 ± 0.2	0.97	0.98	0.99	−0.33	0.01
F1SCC7	LOC396684	Serpin A3-5	31.5 ± 0.2	31.2 ± 0.2	31.0 ± 0.2	0.97	0.98	0.99	0.33	<0.01
F1RLC4	LOX	Protein-lysine 6-oxidase	23.4 ± 0.1	23.5 ± 0.1	23.5 ± 0.1	0.97	0.89	0.99	0.10	0.07
F1S7K2	LRG1	Leucine rich α-2-glycoprotein 1	27.0 ± 0.2	26.6 ± 0.2	26.5 ± 0.2	0.97	0.98	0.99	0.25	<0.01
I3L5Z3	PRG4	Proteoglycan 4	23.4 ± 0.5	22.7 ± 0.4	22.4 ± 0.3	0.97	0.98	0.99	0.36	0.06
F1SGH0	PTPRG	Protein tyrosine phosphatase, receptor type G	22.5 ± 0.4	23.3 ± 0.3	22.4 ± 0.5	0.41	0.76	0.99	0.46	0.01
F1SCD1	SERPINA3-2	A-1-antichymotrypsin 2	27.0 ± 0.7	26.3 ± 0.6	26.6 ± 0.6	0.97	0.65	0.99	−0.61	0.06
TFR1	TFRC	Transferrin receptor protein 1	23.6 ± 0.3	23.9 ± 0.3	24.0 ± 0.3	0.90	0.30	0.99	0.35	0.01

a *Data are 2-based logarithm transformed and shown as mean ± SEM. CON, no antibiotic treatment; PAR, parenteral antibiotics administered; ENT, enteral antibiotics administered*.

b *Regression coefficient from the linear mixed-effect model indicating the effect of NEC severity*.

Among the ECM-related proteins, all 11 proteins showed decreased abundance with increasing NEC severity. Multiple apolipoproteins, including APOA4, APOC2, APOE, APOD, APOC3, ApoN, and proteins related to lipoprotein metabolism (PON1, SAA, RBP4, Transthyretin, PCKS9, PAF-AH, and PLTP) were affected in abundance in response to increasing NEC score. As the NEC score increased, antithrombin III (SERPINC1), PROS1, and factor V decreased, while fibrinogen-α-chain, histidine-rich glycoprotein (HRG), and hyaluronan-binding protein 2 (FSAP), all involved in inflammation-related coagulopathy, showed increased abundance. Abundance of “positive” acute phase proteins, including angiotensinogen, ceruloplasmin, inter-α-trypsin inhibitor, heavy chain H4 (ITI heavy chain H4), lipopolysaccharide-binding protein (LBP), and α-1-antichymotrypsin 2, increased with increasing NEC severity, while “negative” acute phase proteins, including α-2-HS-glycoprotein, albumin, protein AMBP, carboxypeptidase-N-catalytic chain (CPN), ITI heavy chain H2, and transferrin, decreased. Plasma C2, C3, C5a, C6, CFI, and C1inh increased as NEC severity increased, while plasma C1r, C4a, α-, β-, and γ-subunits of C8, CD55, and vitronectin all decreased.

**Figure 3:**
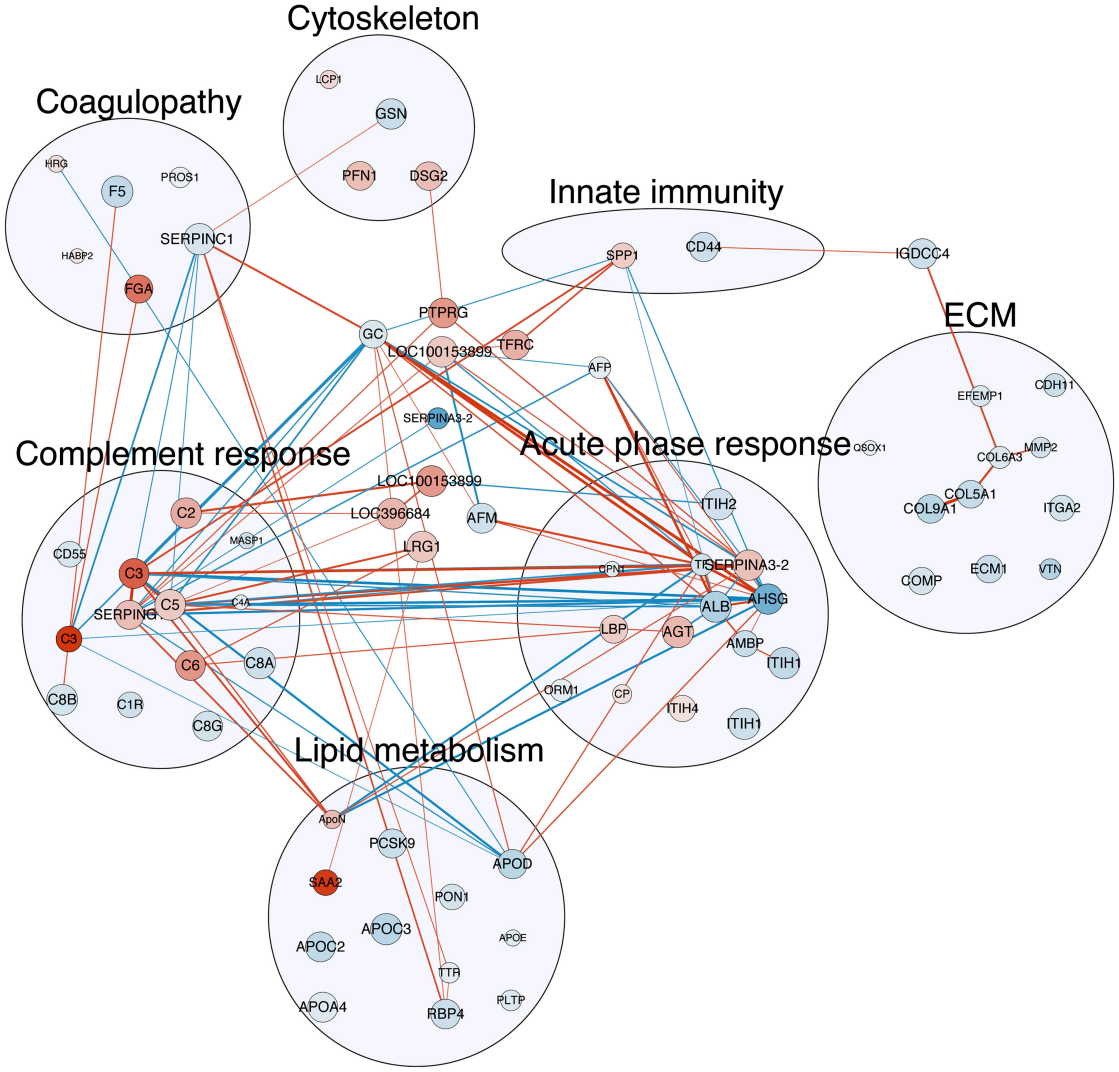
Correlation map of detected plasma proteins with differential abundance with NEC severity. The nodes in red are proteins with increasing abundance with NEC severity, while those in blue are with decreasing abundance. The size of the nodes indicates the q-value of the effect of NEC severity reversely, i.e., the larger the node, the smaller the q-value. The edge (line) in red shows a positive correlation between the two proteins, while those in blue show negative correlation. The width of the edge shows the absolute value of the Spearman's *r*. All correlations listed here are with Spearman's *r* ≥ 0.7. Refer to Table 2 for full protein names.

Multiple correlations were found among the three major protein clusters relating to acute phase response, complement response, and coagulopathy (Figure 3, all Spearman's *r* ≥ 0.7), which, together, constituted the systemic inflammation pertaining to NEC. Besides, correlations were also found between proteins involved in lipid metabolism and the aforementioned three protein clusters (Figure 3, all Spearman's *r* ≥ 0.7), indicating potential interplays between systemic inflammation and lipid metabolism in NEC.

### Gene Expression

**Figure 4:**
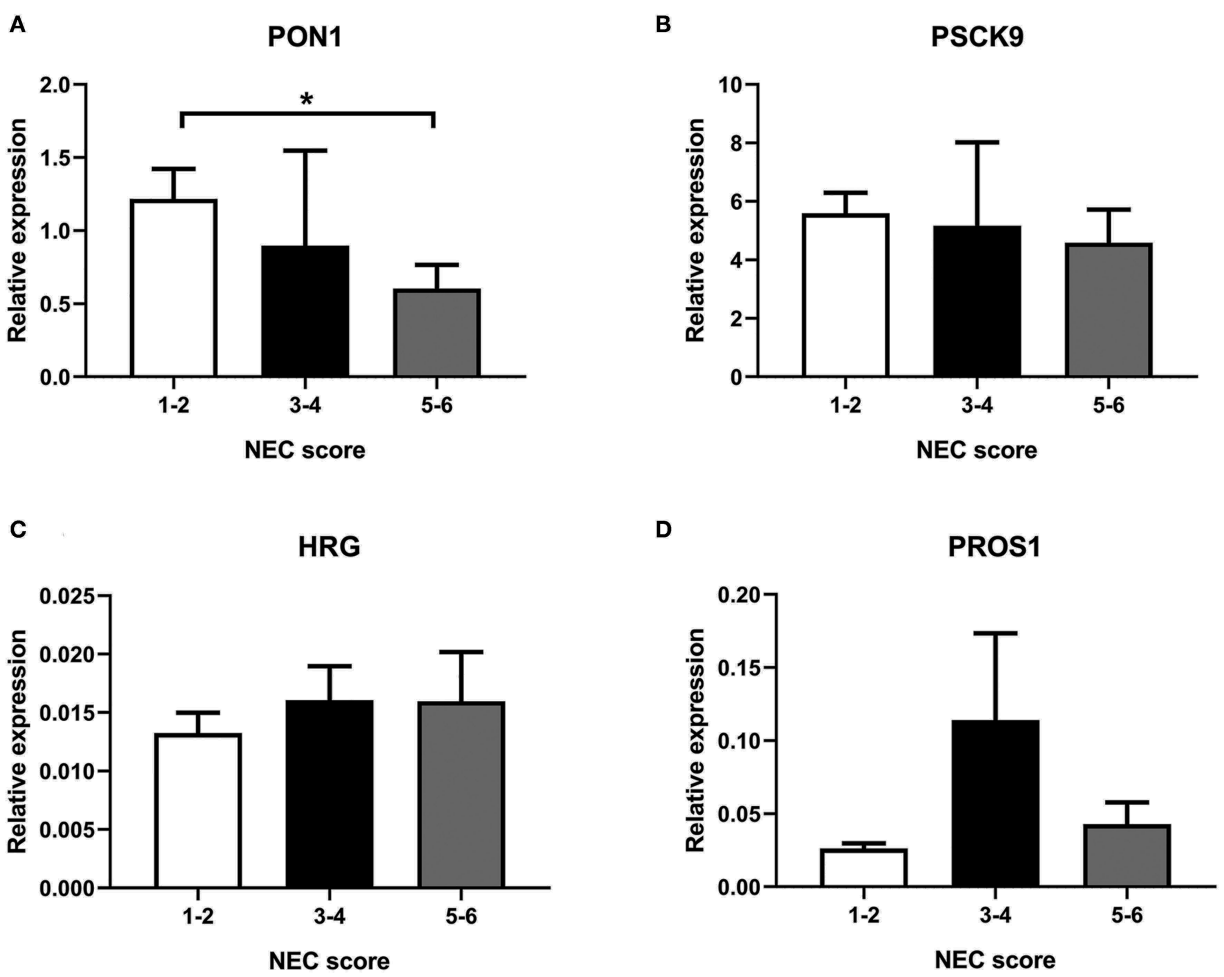
Transcription in the liver of selected genes, **(A)** PON1, **(B)** PSCK9, **(C)** HRG, and **(D)** PROS1. NEC score: 1–2, no-NEC; 3–4, mild NEC; 5–6, severe NEC. Data are presented as mean ± SEM. *P < 0.05.

As shown in Figure 4, transcription of selected genes related to lipid metabolism was tested in the liver. For easier visualization, pigs were grouped into three groups according to their NEC score. Liver PON1 levels tend to decrease in the severe NEC group (*P* < 0.05) (Figure 4A). However, transcription levels of PSCK9, HRG, and PROS1 showed no significant differences among groups with different NEC severity (Figures 4B–D).

### Plasma Abundance and Liver Transcription of CBG

**Figure 5:**
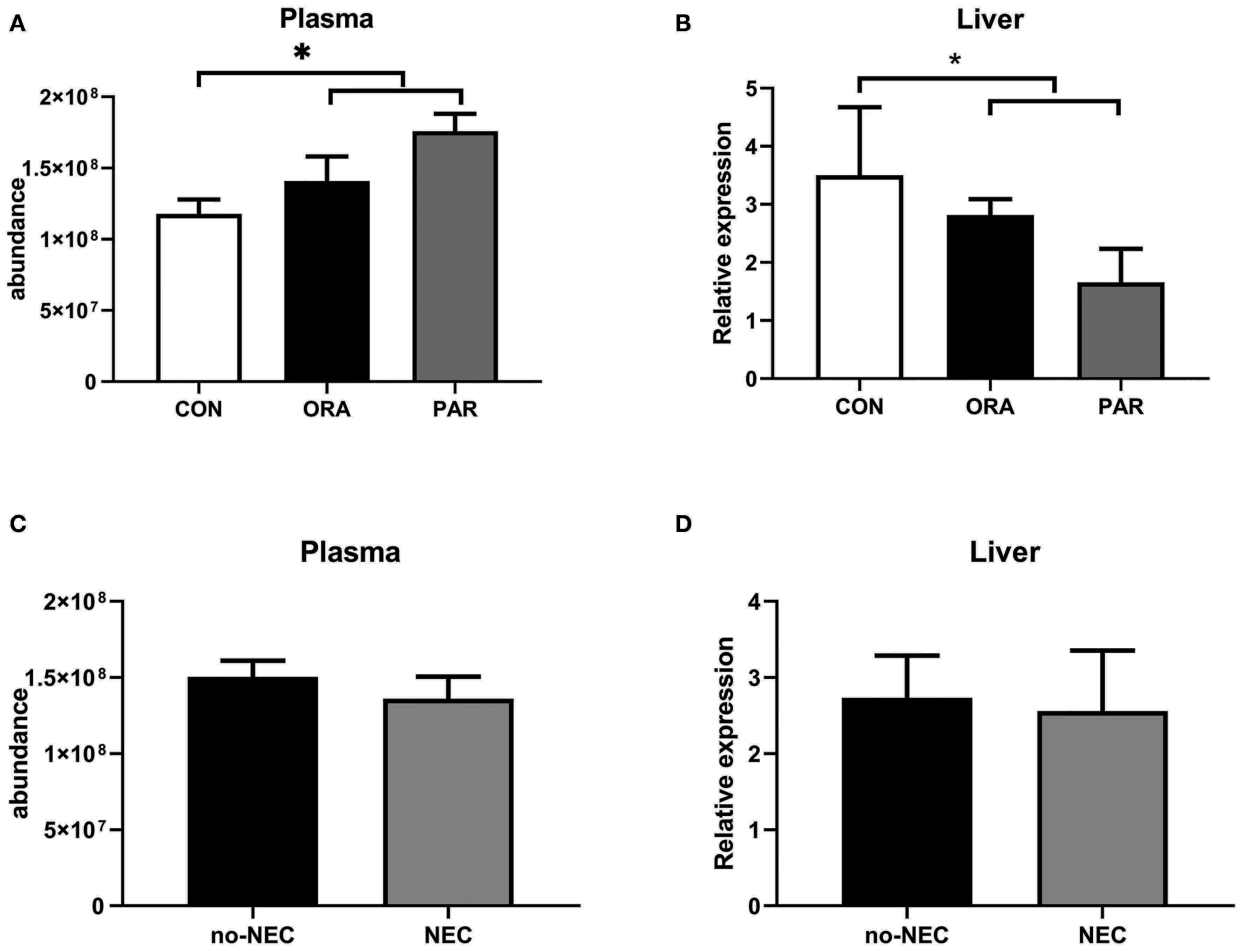
Plasma abundance and transcription levels in the liver of CBG by the antibiotic treatment group **(A,B)** and by the presence of NEC **(C,D)**. Data are presented as mean ± SEM. *P < 0.05. CON, no antibiotic treatment; PAR, parenteral antibiotics administered; ENT, enteral antibiotics administered.

Plasma levels of CBG were significantly higher in the antibiotic-treated groups (both *P* < 0.05) (Figure 5A), while limited effect related to NEC severity was observed (Figure 5C). In contrast to its plasma level, transcription level in the liver of CBG was lower in the antibiotic-treated groups (both *P* < 0.05) (Figure 5B), while no effect of NEC was observed (Figure 5D).

## Discussion

Using preterm pigs as a model for preterm infants, with or without clinically relevant antibiotic treatments, multiple hematological and plasma proteomic markers were affected by NEC severity. In contrast, the antibiotic treatment itself affected much fewer parameters. Due to the fact that the antibiotics, as a treatment for NEC, had a significant effect on NEC scores (*P* < 0.001, linear mixed-effect model, Supplementary Table 1 and Supplementary Figure 1), it is difficult, pathophysiologically, to fully separate the effects of NEC from that of the antibiotic treatment. However, by using NEC scores as continuous data, our statistical analyses showed that NEC severity, not the antibiotic treatment, was the key factor driving changes to plasma proteins. Besides, bacteremia, the presence of bacteria in the blood, may itself trigger changes in plasma proteins (27). In our previous publication on the same set of pigs, bacteremia, detected by blood-agar culture, was documented in CON (9 out of 17) and PAR (2 out of 16) pigs, but was absent in all ENT pigs at euthanasia (16), indicating that NEC in 5 day-old preterm pigs is generally associated with bacteremia. Consequently, it is not possible in this study, like in studies on infants, to separate the plasma proteome effects of NEC lesions in the gut from the effects of NEC-associated systemic inflammation following bacterial translocation. This is similar to the situation in preterm infants with NEC where systemic effects are inevitably the combined result of variable gut lesions, antibiotics treatment, and systemic bacteremia, making it difficult to identify NEC-specific systemic biomarkers.

Among the hematological parameters, absolute cell numbers of neutrophils, lymphocytes, and monocytes, but not their relative proportions, decreased with increasing NEC severity, shown as negative regression coefficients, confirming the observations in infants (3), although no eosinophilia or thrombocytopenia was observed in the pigs. These responses may be partly related to the altered levels of the liver function-related enzymes (ALT, AST, and GGT), representing a joint systemic inflammatory response associated with NEC. Increment in the intestinal permeability found here may have initiated this systemic inflammation by allowing bacteria and their toxins to enter into the circulation. This is underscored by our previous finding of the presence of bacteria in the blood of CON and PAR pigs showing NEC lesions and absence of systemic bacteria in ENT pigs, which were essentially NEC free (16). This bacteremia would, in turn, cause changes in various blood parameters. The observed NEC-associated changes in the blood parameters, including the plasma proteins, may, therefore, be the combined response to microbiota-dependent NEC lesions in the gut and their associated systemic effects in the blood and organs distant to the gut, e.g., liver or kidney.

Disruption of the intestinal ECM, together with intestinal inflammation and immune cell infiltration, is closely associated with NEC pathogenesis (28). Disturbed ECM homeostasis was indicated by a change of a matrix metalloproteinase (MMP-2), an MMP-activating thioredoxin (QSOX1) (29), a product of MMP-mediated cleavage (COL6A3), integrin-α2 and vitronectin (connecting ECM and epithelial cells) and cadherin-11, a cell-adhesion protein. The majority of ECM-associated proteins in plasma were decreased in abundance with increasing NEC severity. However, such proteins may change differently in plasma and in the gut tissue during NEC as intestinal expression of MMP-2,−9, TIMP-1,−2 were reported being elevated in human NEC (30), contrasting our findings in plasma. Similarly, desmoglein-2, a component of desmosome and associated with perturbed epithelial barrier function, increased with increasing NEC severity, but was reduced in the intestinal tissue of patients with IBD (31). In NEC, elevated intestinal expression of ECM-associated proteins, especially MMP-2, −9 and TIMP-1,−2, facilitates the recruitment of immune cells to cross the endothelial and epithelial layers and reach the infection sites. However, inflammation associated with systemic infection and NEC alters the expression of ECM proteins in other organs, too. Similar transcriptional changes of the above proteins have been found in septic rats (32). Thus, it is difficult to attribute changes in such plasma proteins found here to any specific organ due to the ubiquitous expression of these proteins. They may also show an age-related regulation as elevated (not reduced) serum levels of MMP-9 and TIMP-1, as well as reduced MMP-9/TIMP-1 ratio, were observed in adult sepsis (33). While these plasma proteins are of use in early NEC detection, more research is clearly required to examine their utility in differentiating NEC from sepsis.

Altered lipid metabolism and lipoprotein composition are notable in adult infection and inflammation (34), and in neonatal sepsis (35). In neonatal sepsis, plasma levels of total cholesterol, total triglyceride, lipoprotein-a, high-density lipoprotein (HDL), and apolipoprotein A and B are generally reduced, relative to healthy controls (35). HDL composition changes in endotoxemia, and levels of apolipoproteins, such as the main HDL apolipoproteins, apo-A1 and A2, change (35). Similar to the reduced level of ApoA1 and A2 reported in sepsis, plasma levels of ApoC2, C3, and ApoD decreased with increasing NEC severity. A decreased level of PON1, a hydrolytic enzyme associated with HDL (36), was also observed, in agreement with a previous report of infected humans (37). PCKS9, binding to LDLR on the liver and increasing the LDL levels in the circulation (38), decreased in abundance when NEC progressed. Platelet-activating factor acetylhydrolase (PAF-AH) degrades PAF, which is involved in NEC pathogenesis (39). In line with our findings, a lower plasma level of PAF-AH was found in NEC patients (40) and endotoxemic rats (39), while increasing activity of plasma PAF-AH or oral feeding of exogenous PAF-AH protects against NEC (41). Combined, these findings suggest a perturbed lipid metabolism during NEC, either as a cause or a consequence of NEC. However, levels of lipoproteins and other lipid metabolism-related parameters are affected by the regimen of parenteral nutrition. Unlike our pigs, which received parenteral nutrition with identical regimens, regimens of parenteral nutrition for human patients vary profoundly among patients and among clinics, thus the utility of lipid metabolism-related plasma proteins as markers of human NEC requires further investigation.

Coagulopathy, a common systemic feature of NEC (42), is characterized by enhanced coagulation and impaired fibrinolysis (43). Among the proteins observed in this study, antithrombin III from the liver inactivates thrombin and coagulant factors, while PROS1 inhibits coagulation as a cofactor in the inactivation of Factors Va and VIIIa (44). Similar to our findings in NEC, plasma levels of antithrombin III and PROS1 decreased in septic neonates (45). Decreased plasma levels of these two proteins, together with increased levels of fibrinogen-α-chain, may reflect enhanced coagulation in NEC. However, Factor V involved in coagulation showed decreasing abundance, while HABP2, enhancing fibrinolysis, increased moderately with NEC progression. HRG showed an increasing plasma level as NEC increased, but it reportedly decreased in septic mice (46). Some of these affected proteins changed in a different direction for NEC and sepsis (e.g., HRG), but more studies are required to identify NEC- and sepsis-specific biomarkers.

Multiple acute phase proteins, including complement components, were affected by NEC, and in prospective infant studies, plasma inter-α-trypsin inhibitor levels decreased in NEC patients (47). In this study, ITIH1 and ITIH2 (two heavy chains of inter-α-trypsin inhibitor) decreased in abundance, while ITIH4 increased. Most “positive” acute phase proteins increase when NEC progresses, while several “negative” regulators decrease. Detected complement proteins include components from all three major pathways, namely, the classical, alternative, and lectin pathways, and components of the early (C1q, C2, C4a, MASP1), middle (C3, C5a), and late (C6, C8) complement response, together with a receptor (CR1) and inhibitors (CFI, CD55). Among these proteins, AHSG, C3, and C5 were found with a relatively large regression coefficient of NEC. Combined, the complement response, such as increased abundance of C2, C3, C5a, decreased negative regulators, CR1 and CD55, suggests a possibility to detect the early NEC by changes in the complement cascade. A comprehensive study is required to investigate the actions of the complement system in NEC progression to ascertain any potential utility in NEC prediction or detection. Besides, more research is required to show if they are indeed among the earliest systemic signs of NEC progression, when clinical signs are unclear.

Besides the effect to kill or suppress microbes, antibiotics may have both local and systemic anti-inflammatory and vasomodulatory effects (6, 48). Our analyses showed that antibiotics altered blood hematology and biochemistry, such as monocyte counts and albumin levels, with similar effects from the two administration routes (PAR or ENT). Multiple proteins were affected by the antibiotic treatment alone, although corticosteroid-binding globulin (CBG) was also affected by NEC. Levels of CBG, the main cortisol-transporting protein in plasma, decreased during infection and sepsis (49). Lower plasma levels of CBG were found by us in preterm piglets with sepsis (50). In contrast, NEC lesions had limited effect on plasma CBG levels in this study. Similar trends of change at transcription level were found in the liver of these pigs, suggesting that at least part of the systemic CBG change in this study originated from liver effects. The proteomic analyzing technology adopted here can only detect the level of total CBG with no differentiation of the high- or low-affinity types. It is of interest to determine the NEC-related plasma level of high-affinity CBG (haCBG), and its relation to cortisol, as bioactive glucocorticoid levels may play a role in NEC progression and repair.

## Conclusion

In preterm pigs, presence of NEC lesions was associated with numerous systemic plasma protein effects that may be the targets for developing new early biomarkers of NEC. Proteins with large NEC-related changes in abundance (large regression coefficient) were RBP4, FGA, AHSG, C3, C4A, PTPRG, and α-1-antichymotrypsin 2. More research is required to verify their possible utility in indicating NEC in clinical conditions with varying gestational age, antibiotics usage, and feeding regimen, and in differentiating NEC from the conditions inducing systemic effects, but not related to gut complications, including bacteremia and sepsis.

## Data Availability Statement

The datasets presented in this study can be found in online repositories. The names of the repository/repositories and accession number(s) can be found in the article/Supplementary Material.

## Ethics Statement

The animal study was reviewed and approved by The Danish National Committee of Animal Experimentation.

## Author Contributions

Y-NJ contributed to the data analysis and prepared the initial draft. TM conducted the follow-up validation works and contributed to data interpretation. AS conducted the proteomics analysis. DN participated in the animal experiment, prepared the proteomics samples, and contributed to result interpretation. PS conceived the experimental design, contributed to result interpretation, and manuscript preparation. P-PJ contributed to the experimental design, sample processing, data analysis, result interpretation, and manuscript preparation. All authors contributed to the article and approved the submitted version.

## Conflict of Interest

The authors declare that the research was conducted in the absence of any commercial or financial relationships that could be construed as a potential conflict of interest.

## Supplementary Material

Supplementary Figure 1 NEC scores in the treatment groups.

Supplementary Table 1 Macronutrient and mineral content of the parenteral nutrition and formula used.

Supplementary Table 2 NEC scores in the treatment groups. CON, no antibiotic treatment; PAR, parenteral antibiotics administered; ENT, enteral antibiotics administered.

Supplementary Table 3 Primer sequence of selected genes.
