# Supplementary material for: Early Protein Markers of Necrotizing Enterocolitis in Plasma of Preterm Pigs Exposed to Antibiotics
Source: Front Immunol. 2020 Oct 8;11:565862. doi: 10.3389/fimmu.2020.565862 (PMC7578346; doi:10.3389/fimmu.2020.565862)
Supplement: Supplementary Figure 1 — NEC scores in the treatment groups. [file Table_1.DOCX]

**Table S1.** Macronutrient and mineral content of the parenteral nutrition and formula used.

| **Component** | **Content /L** |
| --- | --- |
| **Parenteral nutrition** |  |
| Energy | 3,123 kJ |
| Non-protein energy | 2,373 kJ |
| Glucose | 72 g |
| Lipids | 31 g |
| Nitrogen | 6.4 g |
| Amino acids | 45 g |
| Sodium | 40 mmol |
| Potassium | 28 mmol |
| Magnesium | 3.2 mmol |
| Calcium | 3.2 mmol |
| Phosphate | 12 mmol |
| Osmolality | 1,540 mOsmol |
| **Formula for enteral feeding** | |
| Energy | 4,140 kJ |
| Protein^1^ | 64 g |
| Carbohydrate^2^ | 45 g |
| Fat | 61 g |
| Saturated fat | 44 g |
| Monounsaturated fat | 10 g |
| Polyunsaturated fat | 4 g |
| Sodium | 0.30 g |
| Potassium | 0.64 g |
| Calcium | 0.59 g |
| Phosphorus | 0.42 g |

^1.^mainly whey protein concentrate; ^2.^ mainly glucose.

**Table S2** NEC scores in the treatment groups.

|  | 1~2 | 3~4 | 5~6 | mean ± SEM |
| --- | --- | --- | --- | --- |
| CON | 6 | 2 | 7 | 3.53 ± 0.49 |
| PAR | 7 | 7 | 3 | 2.82 ± 0.40 |
| ORA | 15 | 0 | 0 | 1.33 ± 0.13 |

**Figure S1** NEC scores in the treatment groups.

**, P < 0.01.

**Table S3** Primer sequence of selected genes.

| **Symbol** | **Name** | **Primer Sequences** | |
| --- | --- | --- | --- |
|  |  | Forward | Reverse |
| HPRT1 | Hypoxanthine-guanine  phosphoribosyltransferase (REF) | ACACTGGCAAAACAATGCAA | TGCAACCTTGACCATCTTTG |
| CBG | Corticosteroid-Binding Globulin | ACCCTGTATGCCTGTCTCCT | CAAGGCTGTGGAGATGCTCA |
| PSCK9 | Proprotein convertase subtilisin/kexin type 9 | GTGAAGATGAGCAGCGACCT | CAAGGCGTGGTTTGTTCGAG |
| HRG | Histidine-rich glycoprotein | CTGGGCACTTAAGAAGGCGA | ATGTTTGTGGTGCGGCAATG |
| PROS1 | Protein S | TATCGATCACTCAGCGTGGC | TCCGGCTTAAAAAGGGGTCC |
| PON1 | Paraoxonase 1 | GCTAGTGGTGAGCCATCCAG | AAGTGTTCAGGTCCCACAGC |
| Fetuin-A | Alpha-2-HS-glycoprotein | AGGAGACTGCGATTTCCACG | CTGCAAATAGGAGCCGTTGC |
| Fetuin-B | Fetuin-B | AGCTGCCATGTGCTCAGTAA | TCAGGGCATAGCGTGACAAG |
| CD44 | CD44 | TTCAGCTGTGCCCTCAAACA | GGTGTGGTTGAAATGGTGCT |
| SPP1 | Osteopontin 1 | CACGGGAGACCCCAATGATG | GGGTACCATCCGTCTCCTCA |
| IL-10 | Interleukin 10 | GTCCGACTCAACGAAGAAGG | GCCAGGAAGATCAGGCAATA |
| HIF1A | Hypoxia Inducible Factor 1 Subunit Alpha | TGTGTTATCTGTCGCTTTGAGTC | TTTCGCTTTCTCTGAGCATTC |
| PAF-AH | Platelet Activating Factor- Acetylhydrolase | CCGTGGAATATGAGTGCCGT | AGTTCTCCAGGAGTCGGACA |
| AT-II | Angiotensin 2 receptor | TAACATGCATTGTGGGCAAGC | CGATTGTGGTCAGAGCCCAG |
| TTR | Transthyretin | GGGTGCTGGTGAATCCAAGT | TGCCAAGTGCCTTCCAGTAG |
| RBP4 | Retinol binding protein 4 | TGGTGGGCACCTTTACAGAC | CACGAAGGAGTAGCTGTCGG |
| HABP2 | Hyaluronan Binding Protein2 | AGGACCAAGCTGATCCATGC | CAGCTGGGACCTGTGTAAGG |
| CD55 | CD55 | ACCCAAAGATTCACCACAGC | GAGTGTGGTAAGAGCTGCAATAAGT |
